# Studying mandarin tone sandhi in interaction

**DOI:** 10.1371/journal.pone.0356597

**Published:** 2026-08-19

**Authors:** Eric Pelzl, Yuka Tatsumi, Zhisheng Cui, Navin Viswanathan, Annie J. Olmstead

**Affiliations:** 1 Department of Language Science and Technology, The Hong Kong Polytechnic University, Hong Kong S.A.R, China; 2 Center for Language Science, The Pennsylvania State University, University Park, Pennsylvania, United States of America; 3 Department of Linguistics, Stanford University, Stanford, California, United States of America; 4 Department of Communication Sciences and Disorders, The Pennsylvania State University, University Park, Pennsylvania, United States of America; National Taiwan Normal University, TAIWAN

## Abstract

The Mandarin low tone (T3) undergoes an alteration, third tone sandhi, when followed by another T3. The resulting F0 is superficially the same as that of the rising tone (T2) and has the potential to induce confusion. We investigated how Mandarin speakers adjust their speech production to overcome potential ambiguities induced by third tone sandhi during a structured interactive task. Ten pairs of L1 Chinese participants completed an interactive phrase matching task. Participants were shown displays with Chinese phrases (surname+title). One participant read an indicated phrase which the other selected from their display. There were two conditions. In the no sandhi condition, the title did not induce sandhi, and should result in distinct F0 patterns for the surnames. In the sandhi condition, the title induced third tone sandhi, and should result in homophonous surnames. Task performance showed clear evidence of sandhi-induced ambiguity. Examination of accuracy and tone acoustics suggested that pairs reduced sandhi-induced ambiguity primarily by lowering the F0 of T3 towards the end of its trajectory. We interpret this as avoidance of sandhi application. This study shows that even highly structured interactive tasks have potential to reveal new insights into how speakers and listeners navigate communicative ambiguities caused by tone sandhi. This constitutes an important first step to evaluating how phenomena such as tone sandhi generalize to everyday interactive communication.

## Introduction

In running speech, processes such as coarticulation, assimilation, and sandhi can lead to segmental ambiguity. Consequently, speakers must adjust their speech to cope with ambiguities in a communicative context. A salient example of phonological ambiguity is Mandarin third tone (T3) sandhi [1]. Numerous studies have documented the characteristics of T3 sandhi [2,3] in non-communicative contexts, including its potential to cause ambiguity [4,5]. However, it is unclear how sandhi functions in interaction and what adjustments native Mandarin speakers make in interactive contexts to overcome sandhi-induced ambiguity. The present study investigates whether and how native Mandarin speakers resolve the ambiguities that arise due to T3 sandhi in a highly structured interactive communicative task. By limiting interlocutors’ access to contextual and semantic information, this task isolates disambiguation to the articulatory and acoustic level, providing a window into how speakers and listeners deal with T3 sandhi-induced ambiguity in interaction (for a similar approach to investigating vowel confusions, see [6]).

### Description of T3 Sandhi

Mandarin has four canonical tones. Tone 1 (T1) is a high-level tone. Tone 2 (T2) is a rising tone. Tone 3 (T3) is a low (sometimes a dipping) tone. Tone 4 (T4) is a high-falling tone. These tones are an integral part of words/morphemes in Mandarin, so that changes in tones change meanings, e.g., (T1) ma1 ‘mother’, (T2) ma2 ‘hemp’, (T3) ma3 ‘horse’, (T4) ma4 ‘scold’.

**Fig 1:**
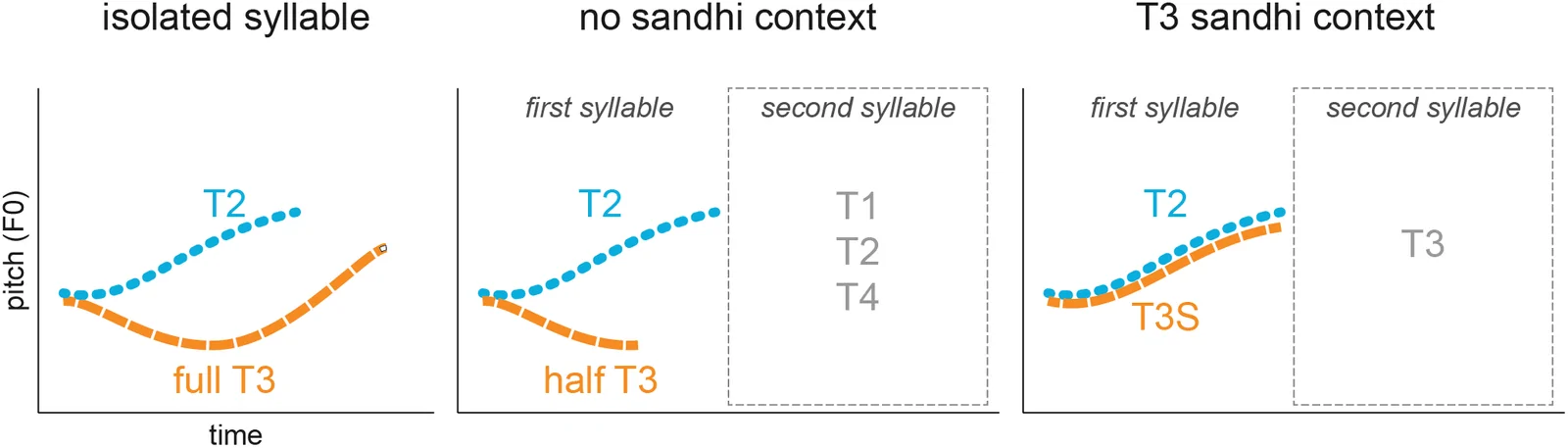
Idealized comparison of Tone 3 F0 contour in different contexts.

While the canonical descriptions above apply quite consistently for tones produced on isolated syllables, the F0 (fundamental frequency) of tones produced in connected speech can be quite different [2]. The most famous example of this is T3, which has three commonly occurring allotonic forms (Fig 1). The “full” dipping form of T3 is often thought of as the canonical form of T3. However, its occurrence is usually restricted to syllables uttered pre-pausally, or in isolation—and even in these cases T3 is not necessarily realized with a full dipping contour [7]. In naturalistic conversations, T3 is most often realized as the so-called ‘half T3’ (or ‘half-third sandhi’ [3]). The half T3 is a low-falling tone, without a subsequent rise. It occurs when T3 precedes any tone other than T3, and can also occur pre-pausally, and in isolation. Finally, there is T3 sandhi. When one T3 is followed by another T3, the first undergoes an alternation so that it is realized as a rising contour. To differentiate this rising sandhi form of T3 from T2, we will refer to it as “T3S”. Importantly, T3S closely resembles T2 (Fig 1). This means that it has the potential to create ambiguity in communication. For instance, after the application of T3 sandhi, the words *fénchǎng* ‘graveyard’ and *fěnchǎng* ‘flour factory’ could sound identical [4,5].

### Previous T3 Sandhi research

While a full account of T3 sandhi research is beyond the scope of this study, we do want to highlight several key findings related to sandhi production and perception (for detailed linguistic analysis, see [3]; for a review of behavioral and neuroimaging studies, see [8]). First, for a simple sequence of two T3 syllables, T3 sandhi is considered to be obligatory. Listeners rate failures to apply sandhi when it is required as less natural than the appropriate T3S [9]. There are exceptions such as when a phrasal break interrupts the sandhi process. Second, T3 sandhi is a spontaneous productive process. In experimental settings, speakers have even been found to apply it to novel T3-T3 ‘wug’ words that they have never encountered previously [3]. Third, while T3 sandhi results in production of a tone (T3S) that is generally assumed to be perceptually indistinguishable from T2, and can result in lexical ambiguity [4,5], there is some evidence that T3S and T2 may—on average—be realized with subtly different acoustic properties. Acoustic studies of T3S produced in laboratory settings have found slightly lower average F0 for T3S compared to T2 [2,10]. Additionally, several large-scale corpus studies have found that T3S and T2 may differ in pitch, duration, and voice quality [11–13]. However, as Yuan and Chen [13] note, even if there are distributional differences between T3S and T2, there is also a great degree of overlap in those distributions. This overlap would suggest that listeners will not be able to rely on any specific cue to disambiguate these tones.

The acoustic and perceptual consequences of T3 sandhi imply that this process would naturally lead to occasional lexical confusions in everyday conversational contexts. Existing corpus studies include natural speech, but have not assessed whether ambiguity occurs. Existing lab-based studies have not examined communicative interactions. It is possible that the production and perception of T3 sandhi in solo contexts provides an accurate description of what Mandarin language users do in interaction. However, past literature examining speech production in interaction cautions us against assuming equivalence without further investigation [14–16].

#### Insights from related domains.

While studies have not examined the production of tones including specifically T3 sandhi in actual interaction, we may look to studies in a related domain for insights as to how interlocutors solve related confusions. One such domain are studies in which interlocutors are tasked with resolving syntactic ambiguity in English. In these studies, researchers often elicit syntactically ambiguous phrases in highly structured interactions through the use of referential communication tasks such as the one we employ in the current study [17,18]. For instance, in one of the earliest investigations of prosodic disambiguation of ambiguous syntax, Schafer and colleagues [17] demonstrated speakers consistently altered their pitch accent, lengthened words at phrase boundaries to disambiguate early vs. late closure sentences that were identical lexically. They played these tokens to a different group of listeners and found that these alterations were helpful in disambiguating these structures on the basis of prosodic adjustments alone. Other studies have demonstrated the systematic use of prosody to signal more pragmatic aspects of discourse such as the introduction of new information, i.e., contrastive focus [19]. For instance, Buxó-Lugo and colleagues [20] examined speakers’ productions of contrastive focus in two types of tasks. One was a non-communicative picture naming that was performed individually and the other was a communicative task that with a partner involved collaborative gameplay to solve a puzzle in the popular game Minecraft). Especially relevant to the current study is their key finding that speakers altered their f0 range to signal new information specifically in the communicative task. Taken together, these studies suggest that interlocutors in actual interaction alter prosodic aspects of the utterance even when other avenues (e.g., lexical alteration) are unavailable to them. Given that these adjustments happen reliably in structured interactions even in a non-tonal language like English, we think that such changes may be observed in our current study with native Mandarin speakers who are faced with tonal ambiguities.

#### Motivation for current study.

In the current study, we investigate ambiguity induced by T3 sandhi, using an experimental paradigm that requires unambiguous communication for task successes, while simultaneously providing tight experimental control over what participants can say. Specifically, we modify the collaborative word matching task that Olmstead and colleagues [6] used to examine tense-lax vowel contrasts in interactions involving Mandarin L1-English L2 bilinguals. That task involved pairs of interlocutors playing a matching game in which, on each trial, one of the participants produced a word highlighted on their screen and their partner picked the correct word from a display on their screen. Participants were not permitted to discuss their choices. Thus, by carefully choosing stimuli, specific, potentially confusing items (e.g., heed vs. hid) were elicited and the resulting acoustic and perceptual patterns examined. Using a similar approach, in the present study we evaluate how T3 sandhi operates in interaction while retaining experimental control through the careful selection of competing tokens on the screen.

We investigated how speakers in an interactive setting navigate the potential for T3 sandhi to induce lexical ambiguities. To do this, we made use of a naturally occurring context in which T3 sandhi ambiguity might arise. In Chinese, surnames precede titles. So, rather than “Director Zhang”, the word order in Chinese would be surname (*Zhang1*) followed by title (*zhu3ren4*, ‘director’). As many Chinese surnames are monosyllabic and differ from similar names only by tone (common examples include Li3 李 vs Li2 黎, Shi3 史vs Shi2 石, and Wu3 武vs Wu2 吳), the potential for confusion is high. Thus, T3 sandhi can produce a situation where a surname that bears T3 might combine with a title that has T3 on its initial syllable, thus producing a T3 sandhi context. For example, a listener hearing “*Lu2 zhu3ren4*” might be unsure if the surname was a rising *Lu2* (卢) or a T3S version of *Lu3* (鲁). Through the systematic manipulation of surnames and titles, we were able to create tone combinations that naturally produced sandhi (or not) in believable contexts. By providing interlocutors with trials wherein the choices are either *Lu2* (卢) or *Lu3* (鲁), the task ensures that interlocutors must address any ambiguities in order to successfully complete the task.

We note that this type of ambiguity is quite plausible in daily interactions due to that fact that the title for ‘teacher’ in Mandarin is *lao3shi1* (teacher’), with T3 on the first syllable. Students almost universally address teachers using this title, and if a teacher’s surname bears T3 and has a T2 competitor, there is a possibility for ambiguity. In fact, when hearing about this project, a colleague reported just this scenario, with students sometimes misconstruing her surname *Xu3* (许) as *Xu2* (徐) due to T3 sandhi-induced ambiguity [Yu-Yin Hsu, personal communication, 6/12/24]. In the present project, we avoided the title *lao3shi1* due to the difficulty it would have caused for locating acoustic transitions between surname offsets and title onsets.

### Research questions

We asked two questions. First, we investigated whether T3 sandhi did in fact result in lexical ambiguities. This would be observed by a drop in accuracy for identification of T3 or T2 surnames in sandhi contexts (titles with T3 on the first syllable) compared to the same surnames in non-sandhi contexts (titles with T1 on the first syllables). Second, we examined whether and how pairs of participants would successfully resolve the ambiguity. We specifically examined whether speakers would adjust their production of T3 (and/or T2) in some way to differentiate surnames when titles supplied a T3 sandhi context. Because we anticipated that individuals and pairs of participants might vary in how they dealt with the sandhi-induced ambiguity, we also augmented these analyses with qualitative exploration of the acoustic adjustments that specific pairs of participants used.

## Methods

### Participants

Twenty L1 Mandarin speakers (10 pairs; 10M/10F; mean age: 24.6; range 20–61) participated in the current study. They were recruited between Nov 3, 2021 and April 18, 2022. Paired participants did not know each other prior to the research session. All participants spoke modern standard Mandarin (Putonghua) as their native language. Questionnaire responses indicated several people also had early knowledge of Chinese dialects or had later learned them. No attempt was made to restrict dialect exposure in the present study. All participants also spoke English as a second language and were in the United States at the time of the study and provided verbal consent to the experimenter to participating in the study. These procedures were reviewed by the Pennsylvania State University’s office for human research protections (Study 11708) and determined that they met the criteria for exempt research.

### Materials

Matching task stimuli consisted of three common Chinese surnames that have the same segmental structure ‘Lu’ (/lu/) but differ in their tonal features. Critically, one of the surnames (*Lu3* 鲁) bears T3, and a second bears T2 (*Lu2* 卢). This allowed us to compare whether T3 differs from T2 when in a sandhi context. A T4 surname (*Lu4* 陆) served as a filler and as a tone that should not be affected by any changes in sandhi context.

Along with the surnames, four disyllabic titles were chosen that contrast in whether they produce a context for tone sandhi (T3 on the first syllable or not): *zhen1tan4* ‘detective’ and *zhu3ren4* ‘director’; *jiang1jun1* ‘general’ and *jing3guan1* ‘police officer’. Pairs of titles were selected so that the onset matched (e.g., *zh* [ʈʂ] in both ***zh****en1tan4* and ***zh****u3ren4*), and so that the tone of the final syllable matched (e.g., T4 in both *zhen1tan****4*** and *zhu3ren****4***). Each surname was combined with each title to create 12 unique stimulus items.

A language background survey was administered at the end of the session. This survey included basic demographic questions, as well as questions about participants’ Chinese language/dialect and second language experience. It also included a series of questions aimed at eliciting whether participants were aware of T3 sandhi and its relevance to the Matching task.

### Procedures

The entire study was carried out online using Zoom and Labvanced [21]. Participants first met with a researcher together in a Zoom room. After orienting them to the study, there was a warm-up activity with each participant reading surnames and titles individually before beginning the Matching task together (afterwards, they also completed an interactive Diapix task, which is not reported here).

The experimental procedure for the Matching task was controlled by Labvanced and audio was recorded simultaneously over Zoom. The task comprised four blocks of 24 trials. In each block, one participant was designated as the “speaker” and one the “listener”. The screen display for the speaker displayed two surname + title phrases. Both phrases shared the same title but had mismatching surnames. For the speaker, one phrase was highlighted indicating that they should produce it out loud. The display for the listener had the same two phrases, but without any indication of which one the speaker produced. The listener’s task was to click on the phrase they believed was produced. After making their choice, both the speaker and the listener were given feedback indicating whether the choice was correct or not. The position of phrases on the screen (left/right) was counterbalanced across trials and participants, so that the phrases the speaker and listener saw were not always in the same locations on their screen. After each block was completed, participants would switch speaker/listener roles. In this way, both participants served twice as speaker, and twice as listener. Cameras were turned off during this task so that participants did not see each other or the researcher. The researcher also monitored to make sure participants did not speak except when producing the words for the Matching task. This disallowed them from discussing or visually negotiating a solution to any ambiguities that arose during the task.

### Data processing

#### Accuracy.

Accuracy data from the Matching task was recorded by Labvanced and processed in R [22]. Correct answers were scored 1; incorrect answers were scored 0. One trial (out of 960) was missing from the data (for Lu2 in the no sandhi context).

#### Acoustic analysis.

Auditory data was extracted from the raw Zoom (.m4a) recordings and imported into Praat [23]. In Praat, we first segmented the unedited audio files into smaller files for each of the experimental tasks (e.g., Matching, Diapix), and then manually added boundaries to mark the onset and offset of each surname + title utterance. We then extracted each of these utterances for further processing and analysis.

The full data set comprised 960 unique surname + title recordings, 96 for each of the 10 participant pairs. Due to human error, one audio file from Pair 2 was lost (a Lu3 sandhi token). We inspected each of the remaining 959 files using auditory and visual cues (waveform and wide-band spectrogram) to manually annotate each of the surnames (excluding the titles) with two tiers, with a third tier of annotation added when required.

The first tier identified the interval from the onset of the first syllable to the beginning of the next syllable, allowing for calculating the duration of the surnames (including any silence before the onset of the title). The surname onset was defined as the point where perturbations in the waveform began at the onset of the /l/ in ‘Lu’. The offset was defined as the onset of the second syllable—specifically, where the perturbations in the waveform became denser and more frequent at the closure of the affricate consonant in the onset of the title (either /ʈʂ/ or /ts/).

The second tier marked the onset and offset of the first syllable where a clean F0 contour could be extracted. In many cases, boundaries were equivalent to those in the first tier, but there were also many exceptions. In cases where the pitch-tracking algorithm in Praat failed to detect pitch values (i.e., there was no visible pitch trajectory), those undetectable points were excluded from the computation of F0 for that file. The boundary was instead marked at the onset/offset of the visible pitch trajectory. Additionally, if the onset displayed visual evidence of a sudden jump up (pitch doubling) or down (pitch halving), the boundary was placed after the jump.

The third tier indicated creaky portions of the first syllable, which were excluded from pitch tracking. During this auditory inspection phase, four tokens were deemed unsuitable for analysis and were excluded: three due to tone mispronunciation (one Lu2 sandhi from pair 3, one Lu2 no sandhi from pair 3, and one Lu4 no sandhi from pair 9, all produced by Speaker B), and one due to background noise (a Lu3 no sandhi token from pair 9 produced by Speaker A).

In the next stage of acoustic analysis, a Praat script extracted F0 values from each surname identified in the second tier, sampling at ten equidistant points across the duration. To optimize F0 tracking accuracy, gender-specific pitch ranges were applied (70–300 Hz for male speakers and 100–450 Hz for female speakers). For each speaker, each token was divided into ten equal sub-intervals, within which multiple pitch measurements were taken (typically at 0.01-second intervals) and averaged to produce a single representative F0 value per sub-interval. When creaky regions or irregular vocal fold vibrations were marked in the third tier, these segments were automatically excluded from analysis. This exclusion (of F0 data points, not entire tokens) affected 7 tokens with Tone 2, 90 tokens with Tone 3, and 41 tokens with Tone 4. These data points were concentrated at latter halves of trajectories, especially for T3 in non-sandhi contexts. This imbalance likely reflects the natural prevalence of creaky voice in Tone 3 [11]. The script also flagged abrupt pitch changes exceeding 30% as potential tracking errors that could be inspected in the final stage of processing.

In the final stage of processing, F0 tracking errors—particularly abrupt pitch changes associated with creaky voice—were manually corrected. This affected nine tokens. Praat was found to underestimate the pitch in these segments, typically reporting values approximately half of the actual F0. To address this, the affected pitch values were manually adjusted by doubling the measured F0. The manually corrected tokens included two Lu3 no sandhi and one Lu3 sandhi token from Pair 1 (Speaker B), two Lu4 no sandhi tokens from Pair 3 (Speaker B), one Lu4 no sandhi token from Pair 6 (Speaker B), one Lu4 no sandhi and one Lu4 sandhi token from Pair 7 (Speaker B), and one Lu4 no sandhi token from Pair 9 (Speaker A).

After these processing stages were completed, a second rater checked the boundaries of a random sample of 10% of the data from each pair (10 of 96 tokens per pair). No serious disagreements were found. Small differences in judgment of boundary placement applied for 11 tokens (differences all less than 10 ms, on average less than +/- 1 ms). One tricky case (difference >20 ms) was reviewed with the first rater and the original boundary was retained.

## Results

### Accuracy

As expected, accuracy was nearly perfect for all trials in the no sandhi condition (m = 99%, sd = 1%). For trials in sandhi context, however, descriptive statistics suggest a strong effect for trials with ambiguous competitors (Table 1). That is, when the target Lu3 was produced in a sandhi context and the competitor on the display was Lu2, accuracy dropped. The same was true for target Lu2 with competitor Lu3. In contrast, when the target or competitor was Lu4, accuracy was at ceiling.

**Table 1:** Accuracy in the matching task.

Target	Competitor	ACC (%)	SD
**Lu2**	**Lu3**	76	43
	Lu4	99	11
**Lu3**	**Lu2**	71	46
	Lu4	100	0
Lu4	Lu2	100	0
	Lu3	100	0

This table displays percent correct matching trials for all target competitor pairs in sandhi context. Bolding indicates pairs in which T3 sandhi context will likely create ambiguity.

Statistical analyses were conducted in R using the afex [24] and emmeans [25] packages. Filler trials with Lu4 as target were removed prior to analysis to avoid modelling problems caused by perfect accuracy in the data. The remaining accuracy data were submitted to a by-subjects repeated measures analyses of variance (RM ANOVA) with the factors surname (Lu2, Lu3), and competitor condition (ambiguous, unambiguous). There was no effect of surname (F(1,19)=0.16, η_p^2  = .008, p = .691), a significant effect of competitor condition (F(1,19)=48.47, η_p^2  = .718, p < .001), and no interaction (F(1,19)=0.50, η_p^2  = .026, p = .489), indicating that, regardless of whether the target was Lu2 or Lu3, accuracy in sandhi context dropped when an ambiguous competitor was present.

### Acoustic analysis

**Fig 2:**
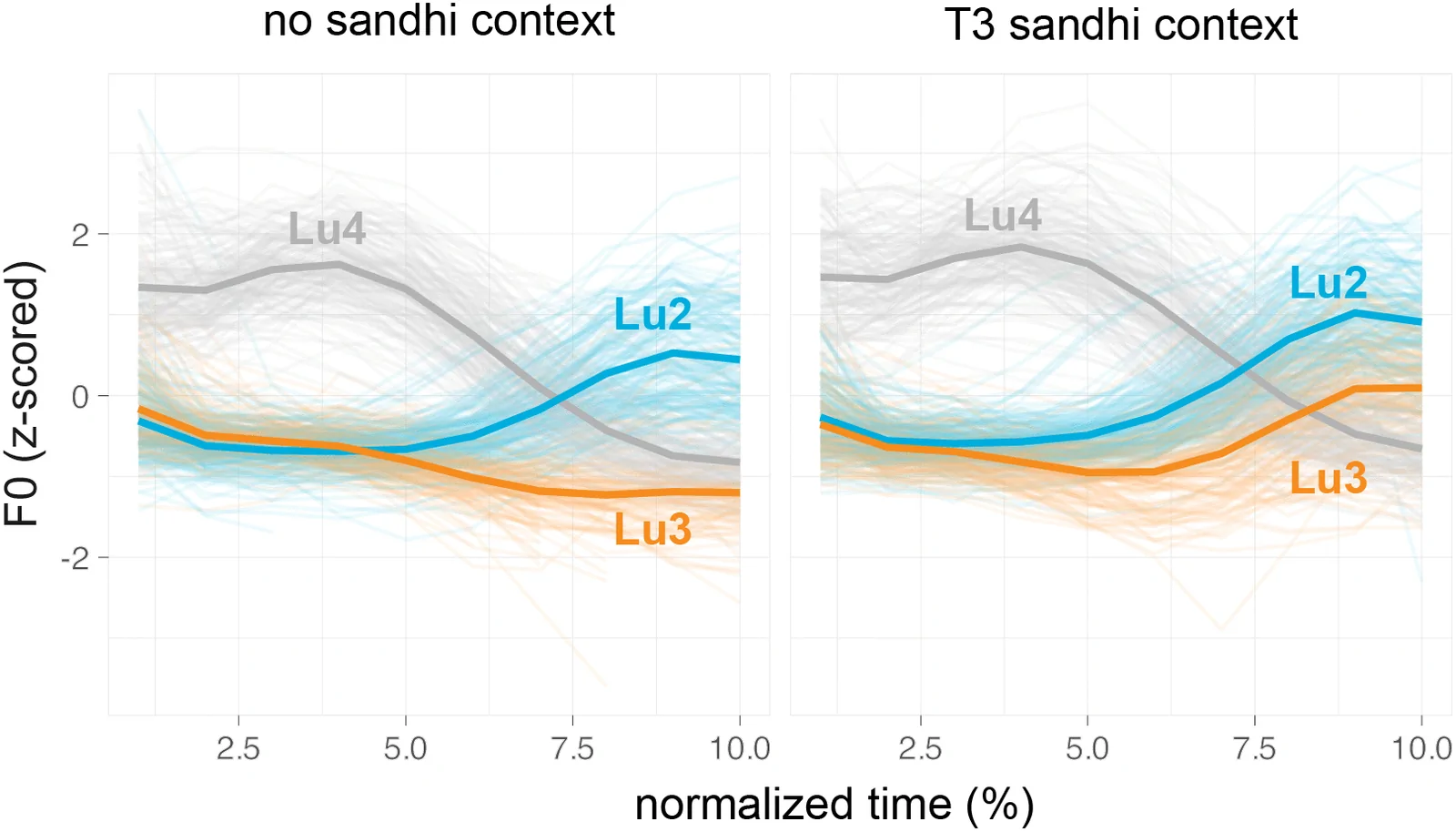
Average raw F0 trajectories with normalized time. Group average is displayed as thick lines. Individual participant trajectories are displayed as thin lines.

Average F0 trajectories were extracted for each surname in each context. There were clear visual differences in the average F0 for Lu3 depending on context, indicating a low-falling trajectory in the no sandhi context, and a low-dipping trajectory before sandhi-inducing titles (Fig 2).

F0 trajectories were modelled using generalized additive mixed models (GAMMs; [26]; for useful tutorials, see [27–29]) using the bam() function from the mgcv package [26], and further analyzed using functions from the itsadug package [30]. We used Claude (Anthropic, Claude 4.7 Opus) to assist with model comparisons and diagnostics, and to generate related R code. All analyses, decisions, and conclusion are our own. GAMMs build on standard regression models by allowing non-linear shapes to occur in time-series data. This is accomplished by utilizing regression smooths that can independently model the wiggliness of each series of time points in the data. Similar to linear mixed-effects models, they also allow for modeling of random effects (e.g., of participants and items). Data, R code, and a written summary of model diagnostics can be found at the OSF link provided at the end of this report.

We fit GAMMs to the within-speaker normalized F0 values (F0_z) sampled from 10 equally spaced timepoints for each surname produced during the Matching task. We included a parametric term for the variables Surname (Lu2, Lu3, Lu4) and Sandhi context (no sandhi, sandhi), coded as an interaction term “Tone.Sandhi”, and also the interaction with centered duration of the surname (Duration_c). We included the reference smooth s(Time) to capture the average F0 contour across all conditions, and s(Time, by = Tone.Sandhi, k = 10) which estimates how the F0 contour deviates from the reference for each level of Tone.Sandhi. This provides the central test of tone-by-Sandhi-context effects on F0 trajectories.

Two tensor-product interactions modeled the influence of syllable duration on the F0 trajectory: ti(Time, Duration_c, k = 10) captures the average modulation of the contour by duration, and ti(Time, Duration_c, by = Tone.Sandhi, k = 10) allows this duration-by-time surface to differ across tone and sandhi contexts.

Trial-position effects on the F0 trajectory were modeled with ti(Time, BlockTrial, k = c(10, 5)) and a corresponding by-Tone.Sandhi interaction ti(Time, BlockTrial, by = Tone.Sandhi, k = c(10, 5)). Note: A marginal s(BlockTrial) smooth was also tested; it did not meaningfully improve fit (ΔfREML = 0.007; ΔAIC = 1.78), and was omitted in the interest of parsimony.

The random-effects structure consisted of two terms. A factor smooth s(Time, Subject, bs = “fs,” m = 1, k = 10) captures subject-specific deviations from the population-level F0 contour [31]. A by-trial random intercept s(FileName, bs = “re”)accounts for unmodeled variation at the trial level.

All smooths used thin-plate regression splines (the default basis in *mgcv*). With ten F0 points in the data, the maximal number of basis functions was 10. The final model also accounted for autocorrelation with an AR(1) parameter.

The full model specification and summary results are included in Table 2. Results indicated that each surname in both sandhi and no sandhi context differed significantly from the reference level (all ps < .001).

**Table 2:** GAMM summary for all surnames (overall GAMM).

A. Model specification				
f0_z ~ Tone.Sandhi * Duration_c				
+ s(Time)				
+ s(Time, by = Tone.Sandhi, k = 10)				
+ ti(Time, Duration_c, k = 10)				
+ ti(Time, Duration_c, by = Tone.Sandhi, k = 10)				
+ ti(Time, BlockTrial, k = c(10, 5))				
+ ti(Time, BlockTrial, by = Tone.Sandhi, k = c(10, 5))				
+ s(Time, Subject, bs = “fs”, m = 1, k = 10)				
+ s(FileName, bs = “re”)				
B. Parametric coefficients				
	Estimate	Std. Error	t value	Pr(>|t|)
(Intercept)	-0.200	0.024	-8.222	<.001
Tone.SandhiT3.No	-0.666	0.036	-18.720	<.001
Tone.SandhiT4.No	0.719	0.036	19.925	<.001
Tone.SandhiT2.Yes	0.270	0.034	7.903	<.001
Tone.SandhiT3.Yes	-0.241	0.036	-6.612	<.001
Tone.SandhiT4.Yes	0.939	0.037	25.132	<.001
Duration_c	0.002	0.000	5.150	<.001
Tone.SandhiT3.No:Duration_c	-0.002	0.001	-4.672	<.001
Tone.SandhiT4.No:Duration_c	-0.003	0.001	-4.748	<.001
Tone.SandhiT2.Yes:Duration_c	0.000	0.001	0.239	.811
Tone.SandhiT3.Yes:Duration_c	-0.004	0.000	-7.184	<.001
Tone.SandhiT4.Yes:Duration_c	-0.003	0.001	-6.060	<.001
C. Approximate significance of smooth terms				
	edf	Ref.df	F	p-value
s(Time)	8.322	8.430	55.322	<.001
s(Time):Tone.SandhiT3.No	8.341	8.855	236.396	<.001
s(Time):Tone.SandhiT4.No	8.797	8.964	765.161	<.001
s(Time):Tone.SandhiT2.Yes	5.196	6.489	7.298	<.001
s(Time):Tone.SandhiT3.Yes	5.274	6.543	13.353	<.001
s(Time):Tone.SandhiT4.Yes	8.792	8.964	829.590	<.001
ti(Time, Duration_c)	30.159	40.137	4.635	<.001
ti(Time, Duration_c):Tone.SandhiT3.No	9.828	14.577	2.913	<.001
ti(Time, Duration_c):Tone.SandhiT4.No	32.337	44.136	6.362	<.001
ti(Time, Duration_c):Tone.SandhiT2.Yes	11.406	15.268	1.595	.070
ti(Time, Duration_c):Tone.SandhiT3.Yes	13.359	19.084	4.868	<.001
ti(Time, Duration_c):Tone.SandhiT4.Yes	26.887	36.395	7.929	<.001
ti(BlockTrial, Time)	8.035	10.829	4.128	<.001
ti(BlockTrial, Time):Tone.SandhiT3.No	9.438	12.878	2.455	.003
ti(BlockTrial, Time):Tone.SandhiT4.No	11.129	15.115	4.792	<.001
ti(BlockTrial, Time):Tone.SandhiT2.Yes	1.001	1.001	5.033	.025
ti(BlockTrial, Time):Tone.SandhiT3.Yes	7.444	9.945	4.395	<.001
ti(BlockTrial, Time):Tone.SandhiT4.Yes	11.595	15.399	4.819	<.001
s(Time, Subject)	157.414	198.000	12.824	<.001
s(FileName)	686.209	943.000	2.698	<.001

R-sq.(adj) = 0.891 Deviance explained = 90.4%

fREML = 1196.5 Scale est. = 0.083193 *n* = 9218

This GAMM modeled the interaction of surnames (T2, T3, T4) and context (with or without a title bearing T3 on its first syllable; Yes = sandhi context; No = no sandhi context) and the interaction of Duration (centered).

**Fig 3:**
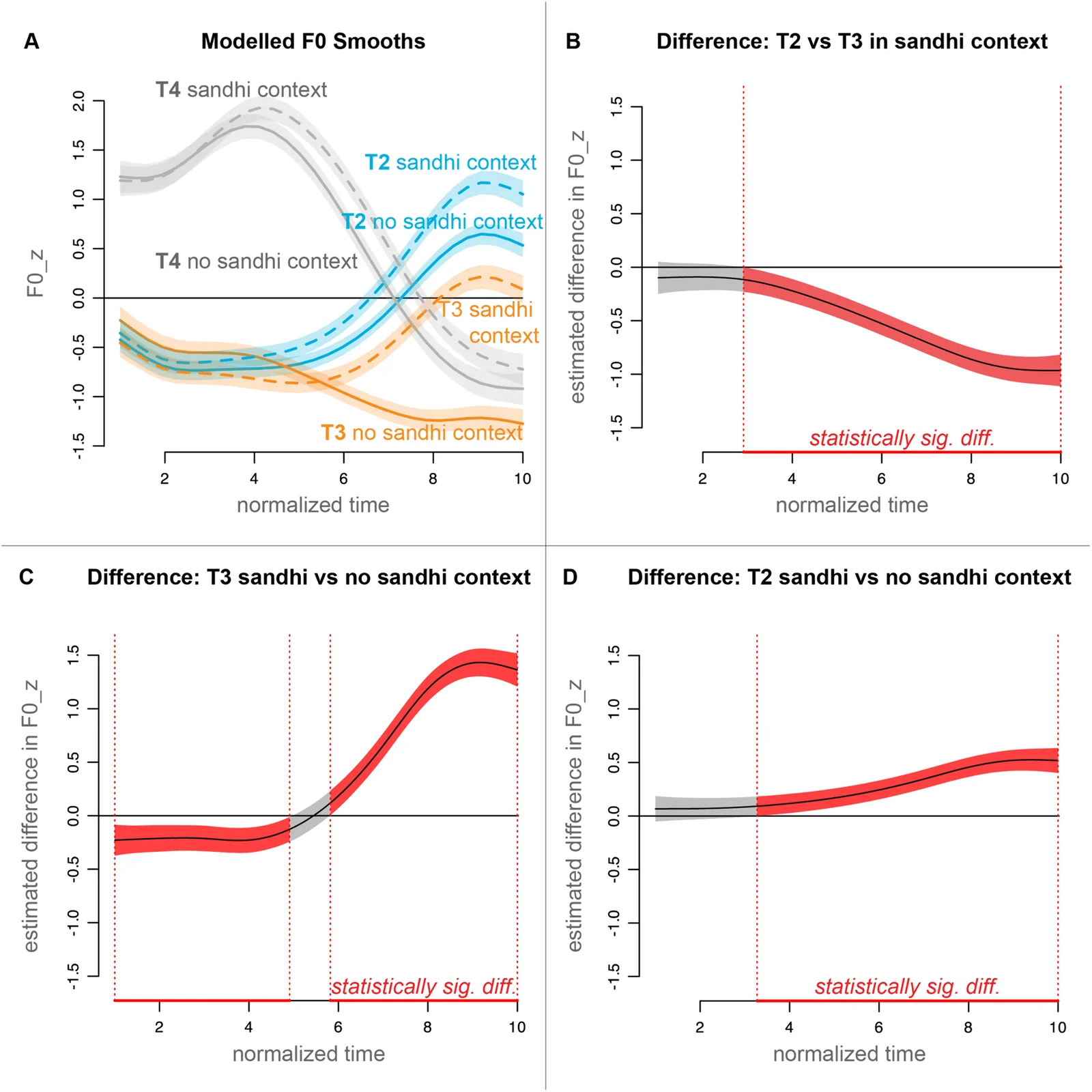
GAMM smooths marginalized over random effects for all tones (Panel A) and difference smooths for critical comparisons (Panels B, C, and D). 95% CI shaded.

In order to further investigate model outcomes, we examined plots of the continuous GAMM smooths. Fig 3 (Panel A) depicts the modeled F0 smooths of each of the surnames in no sandhi and sandhi context (i.e., before titles that either would or would not typically induce T3 sandhi). We also examined difference smooths for the three contrasts of critical interest: the difference between Lu2 and Lu3 in sandhi context, and the difference between contexts for both Lu2 and Lu3. In the first case, relative to Lu2, Lu3 had an overall lower F0 trajectory beginning at about the 3rd time point and continuing for the rest of the trajectory (Fig 3, panel B). When comparing Lu3 between contexts, if a title supplied a sandhi context, Lu3 had significantly lower F0 from onset to about the 5th timepoint, and a significantly higher F0 from roughly the 6th timepoint through to the end of the trajectory (Fig 3, panel C). When comparing Lu2 between contexts, if a title supplied a sandhi context, Lu2 had significantly higher F0 from roughly the 3rd timepoint through to the end of the trajectory (Fig 3, panel D).

In order to specifically evaluate the impact of ambiguity on participants’ F0 trajectories, we also fit GAMMs to the subset of acoustic data where ambiguous competitors were present, that is, where listeners’ identification accuracy dropped. The second model exactly parallels the structure of the overall GAMM.

We included a parametric term for the variables Surname (Lu2, Lu3) and correctness of response (correct, incorrect), coded as an interaction term “Amb.Correct”, and also the interaction with centered duration of the surname (Duration_c).. We included a reference smooth s(Time) to captures the average F0 contour across all conditions, and s(Time, by = Amb.Correct, k = 10) which estimates how the F0 contour deviates from the reference for each level of Amb.Correct.

Two tensor-product interactions modeled the influence of syllable duration on the F0 trajectory: ti(Time, Duration_c, k = 10) captures the average modulation of the contour by duration, and ti(Time, Duration_c, by = Amb.Correct, k = 10) allows this duration-by-time surface to differ across tone conditions.

Trial-position effects on the F0 trajectory were modeled with ti(Time, BlockTrial, k = c(10, 5)) and a corresponding by-Amb.Correct interaction ti(Time, BlockTrial, by = Amb.Correct, k = c(10, 5)).

The random-effects structure consisted of two terms. A factor smooth s(Time, Subject, bs = “fs,” m = 1, k = 10) and a by-trial random intercept s(FileName, bs = “re.”

All smooths used thin-plate regression splines. The final model also accounted for autocorrelation with an AR(1) parameter.

The model summary is included in Table 3. Results indicated that T3 with both correct and incorrect responses differed significantly from the reference level (all ps < .01), while T2 with incorrect responses did not differ significantly from the reference level (T2 with correct responses).

**Table 3:** GAMM summary for ambiguous surnames (ambiguity GAMM).

A. Model specification				
f0_z ~ Amb.Correct * Duration_c				
+ s(Time)				
+ s(Time, by = Amb.Correct, k = 10)				
+ ti(Time, Duration_c, k = 10)				
+ ti(Time, Duration_c, by = Amb.Correct, k = 10)				
+ ti(Time, BlockTrial, k = c(10, 5))				
+ ti(Time, BlockTrial, by = Amb.Correct, k = c(10, 5))				
+ s(Time, Subject, bs = “fs”, m = 1, k = 10)				
+ s(FileName, bs = “re”)				
B. Parametric coefficients				
	Estimate	Std. Error	t value	Pr(>|t|)
(Intercept)	0.035	0.039	0.914	.361
Amb.CorrectT2.0	-0.045	0.094	-0.480	.631
Amb.CorrectT3.0	-0.343	0.069	-5.005	<.001
Amb.CorrectT3.1	-0.588	0.061	-9.562	<.001
Duration_c	0.002	0.001	3.248	.001
Amb.CorrectT2.0:Duration_c	0.000	0.001	0.277	.782
Amb.CorrectT3.0:Duration_c	-0.003	0.001	-2.677	.008
Amb.CorrectT3.1:Duration_c	-0.003	0.001	-3.947	<.001
C. Approximate significance of smooth terms				
	edf	Ref.df	F	p-value
s(Time)	8.279	8.473	67.401	<.001
s(Time):Amb.CorrectT2.0	1.000	1.000	2.209	.137
s(Time):Amb.CorrectT3.0	4.153	5.284	14.907	<.001
s(Time):Amb.CorrectT3.1	6.040	7.293	55.613	<.001
ti(Time, Duration_c)	19.796	26.523	4.236	<.001
ti(Time, Duration_c):Amb.CorrectT2.0	1.000	1.000	0.089	.766
ti(Time, Duration_c):Amb.CorrectT3.0	4.059	6.067	2.566	.018
ti(Time, Duration_c):Amb.CorrectT3.1	3.398	4.990	0.674	.640
ti(BlockTrial, Time)	1.000	1.000	0.685	.408
ti(BlockTrial, Time):Amb.CorrectT2.0	1.000	1.000	3.135	.077
ti(BlockTrial, Time):Amb.CorrectT3.0	8.837	11.957	2.296	.006
ti(BlockTrial, Time):Amb.CorrectT3.1	8.446	11.264	4.323	<.001
s(Time, Subject)	131.785	198.000	3.942	<.001
s(FileName)	111.505	153.000	2.974	<.001

R-sq.(adj) = 0.853 Deviance explained = 88.3%

fREML = 172.37 Scale est. = 0.063833 n = 1582

This GAMM modelled the interaction between T2 and T3 surnames and the accuracy of listeners’ responses, coded as the interaction term “Amb,Correct”. 1 = correct response; 0 = incorrect response.

**Fig 4:**
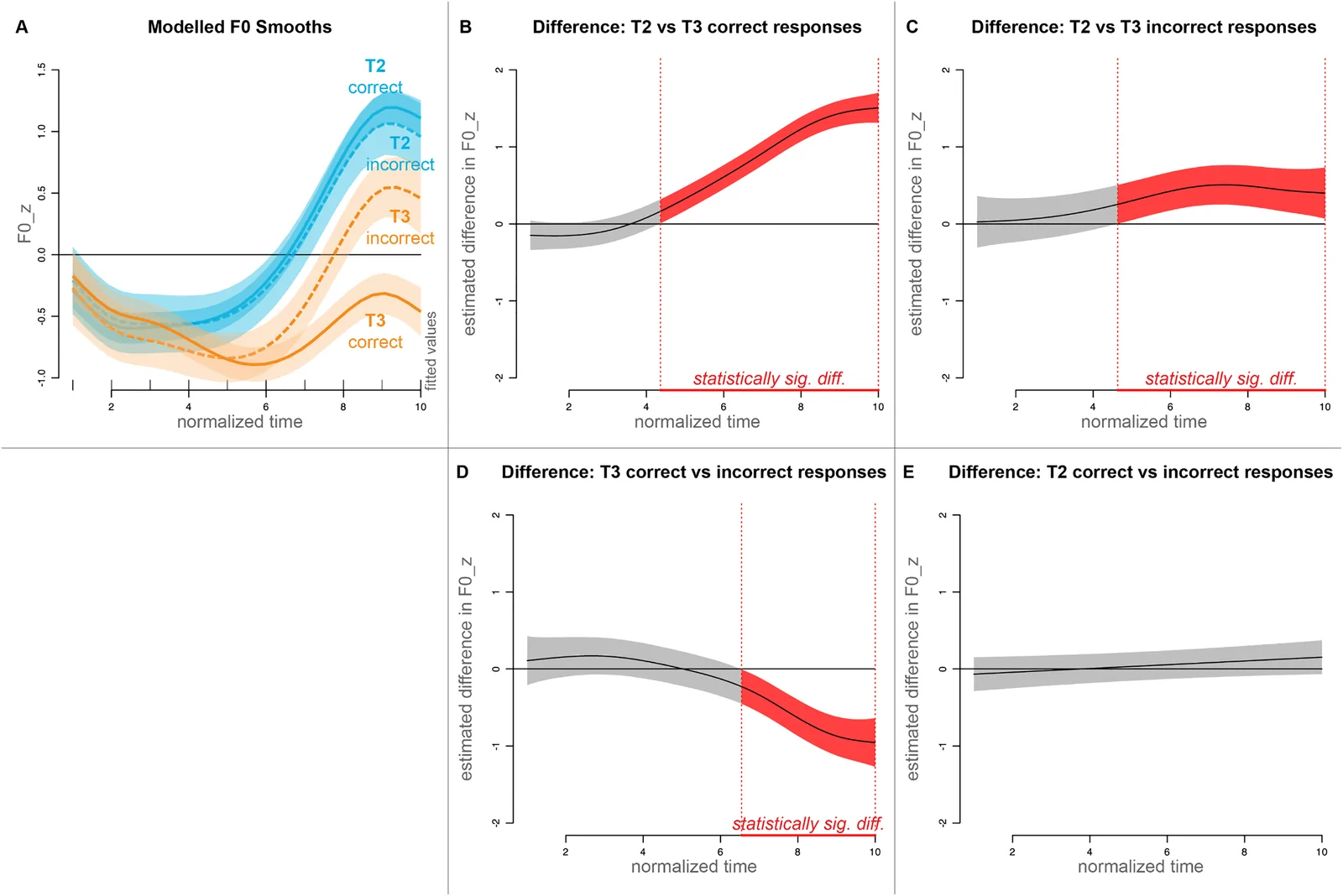
GAMM smooths marginalized over random effects for Lu2 (solid lines) and Lu3 (dashed lines) with correct and incorrect responses (Panel A) and difference smooths for critical comparisons (Panels B, C, D, and E). 95% CI shaded.

Once again, we examined plots of the continuous GAMM smooths. Fig 4 (Panel A) depicts the modeled F0 smooths of each of Lu2 and Lu3 according to whether they were correctly identified when an ambiguous competitor was present on the screen. We also examined difference smooths for the four contrasts of critical interest, comparing Lu2 and Lu3 with correct responses, Lu2 and Lu3 with incorrect responses, and comparing correct and incorrect responses for Lu2 and Lu3 respectively. In the first case, relative to Lu3, Lu2 with correct responses had an overall higher F0 trajectory beginning at about the 4th time point and continuing for the rest of the trajectory (Fig 4, panel B). Again, relative to Lu3, Lu2 with incorrect responses had an overall higher F0 trajectory beginning at about the 5th time point and continuing for the rest of the trajectory (Fig 4, panel C), but with much smaller magnitude of difference compared to the surnames with correct responses. When comparing Lu3 with correct vs incorrect responses, with correct responses the F0 was lower from after the 6th timepoint to the end (Fig 4, panel D). When comparing Lu2 with correct vs incorrect responses, with correct responses the F0 was not significantly different at any timepoint (Fig 4, panel E). In summary, there were significant F0 differences between T3 and T2 regardless of identification accuracy, and between correctly and incorrectly identified T3s, but not between correctly and incorrectly identified T2s.

### Qualitative exploration of acoustic data

Although the quantitative GAMM analysis provides clear evidence of differences in how speakers produced Lu2 and Lu3 during the Matching task, relying only on the group averages obscures some of the observable acoustic patterns in data from individual speakers/pairs. In this section, we briefly explore a few illustrative examples to highlight how Lu2 and Lu3 were often produced in sandhi context. As we will see, there is qualitative evidence that the ‘average’ Lu3 tone in the overall GAMM analysis is an artefact of meaningful variation that occurred when speakers applied T3 sandhi (resulting in T3S), or took steps to avoid ambiguity by non-application of T3 sandhi (resulting in a half T3 or full T3). We will also provide some supporting evidence that was elicited in the post-experiment questionnaire. In this exploratory analysis, we are not claiming generalizability of findings. Instead, our hope is to identify patterns that might be targeted in future research that tests these trends quantitatively.

To guide our qualitative analysis, first, we identified pairs that made more or fewer errors of surname identification for critical trials. To do this, we averaged each pair’s accuracy across T2 and T3 surnames with ambiguous competitors in sandhi context. For example, Pair 1 identified 69% (11/16) T2/T3 surnames correctly. Among all pairs, Pair 6 had the highest accuracy (94%), and Pair 9 had the lowest accuracy (44%).

**Fig 5:**
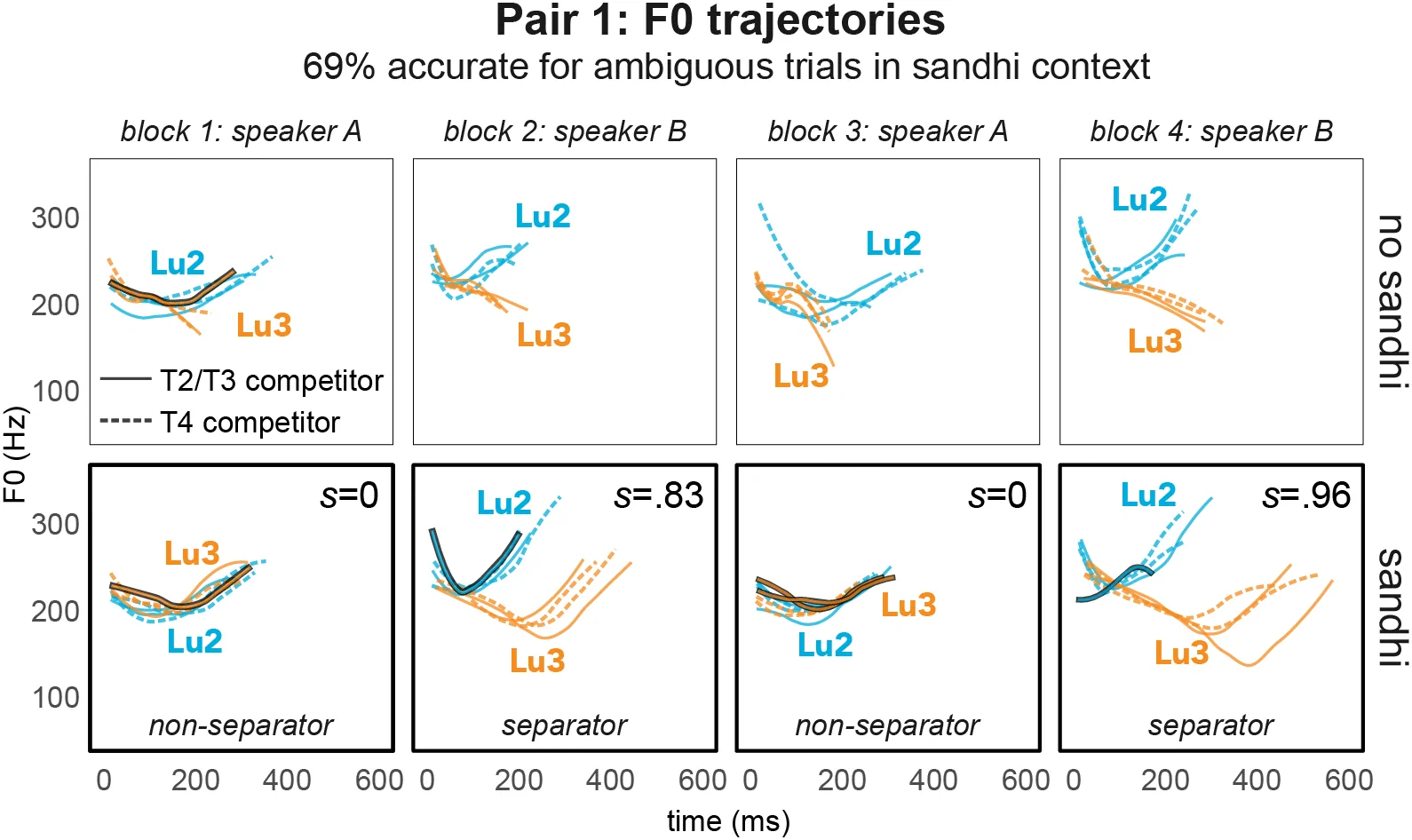
F0 trajectories for Lu2 and Lu3 in different sandhi contexts produced by Pair 1 across blocks of the Matching task. Misidentified tokens have a black outline.

Next, we created a qualitative *separation index* for productions in sandhi context for each speaker for each block. First, we classified each individual T3 trajectory in the sandhi context as ‘separated’ or not, based on whether the latter portion of a T3 trajectory overlapped with any T2 trajectories from the same speaker in the same block. In this manner, from 0–4 of the T3 trajectories could be labelled as separated. Second, for trajectories that were separated, three raters independently gave a subjective rating of the degree of separation for the T3 trajectory compared to the nearest T2 trajectory from the same speaker in the same block. Ratings were ‘low’, ‘medium’, or ‘high’ separation (scored 0, 0.5, and 1 respectively). Raters first examined the visualizations from all pairs to establish a sense for the range of possibilities, then assigned their ratings to individual pairs. We then multiplied the number of separated trajectories (from 0 to 4) for each speaker in each block by the average separation score for the trajectories in that block. This gave us the ‘separation index’ (*s*) for each speaker’s T2 and T3 trajectories in each block. We then classified each speaker in each block as a ‘separator’ (*s* ≥ .66), ‘non-separator’ (*s* ≤ .33), or neither (*s* between .33 and .66). This approach is illustrated with the trajectories from Pair 1 in Fig 5. For this pair, Speaker A produced T3 such that it always overlapped with T2, while Speaker B produced T3 with considerable visual separation from T2. The similarity index is provided in the upper right corner of each plot for sandhi context, and the speaker is labelled accordingly.

Using this method, we can identify 9 blocks with speakers who performed as separators, who appear to be utilizing F0 to create distance between T2 and T3 surnames. On the other hand, there are 17 blocks with speakers who performed as non-separators, who appear not to be utilizing F0 to distinguish T2 and T3. These speakers represent the extremes of the variation that we noted above. Pair 1 (Fig 5) nicely illustrates this bimodal pattern for T3 realizations, as Speaker A is a consistent non-separator, producing very similar T3 and T2 tokens, suggesting consistent application of T3 sandhi; in contrast, Speaker B is a consistent separator, strongly distinguishing T3 from T2, suggesting non-application of T3 sandhi. Pair 1 also illustrates that the relationship between separators and accuracy is not straightforward as there are identification errors in all blocks, regardless of whether the speaker is separating tones or not. This indicates a limitation of visual only analysis, as other features such as voice quality or pauses are not readily available for inspection.

Based on consideration of both accuracy and the presence of separators, we chose two pairs to highlight for illustrative purposes below. Pair 9, which had low accuracy (44%) and one non-separator, and Pair 4 which had intermediate accuracy (88%) with two strong separators. Where appropriate, we will also note where patterns we identify from these pairs are repeated by other pairs or speakers. Figures of trajectories for all pairs not shown here are presented in the Supporting Information at the end of this paper.

**Fig 6:**
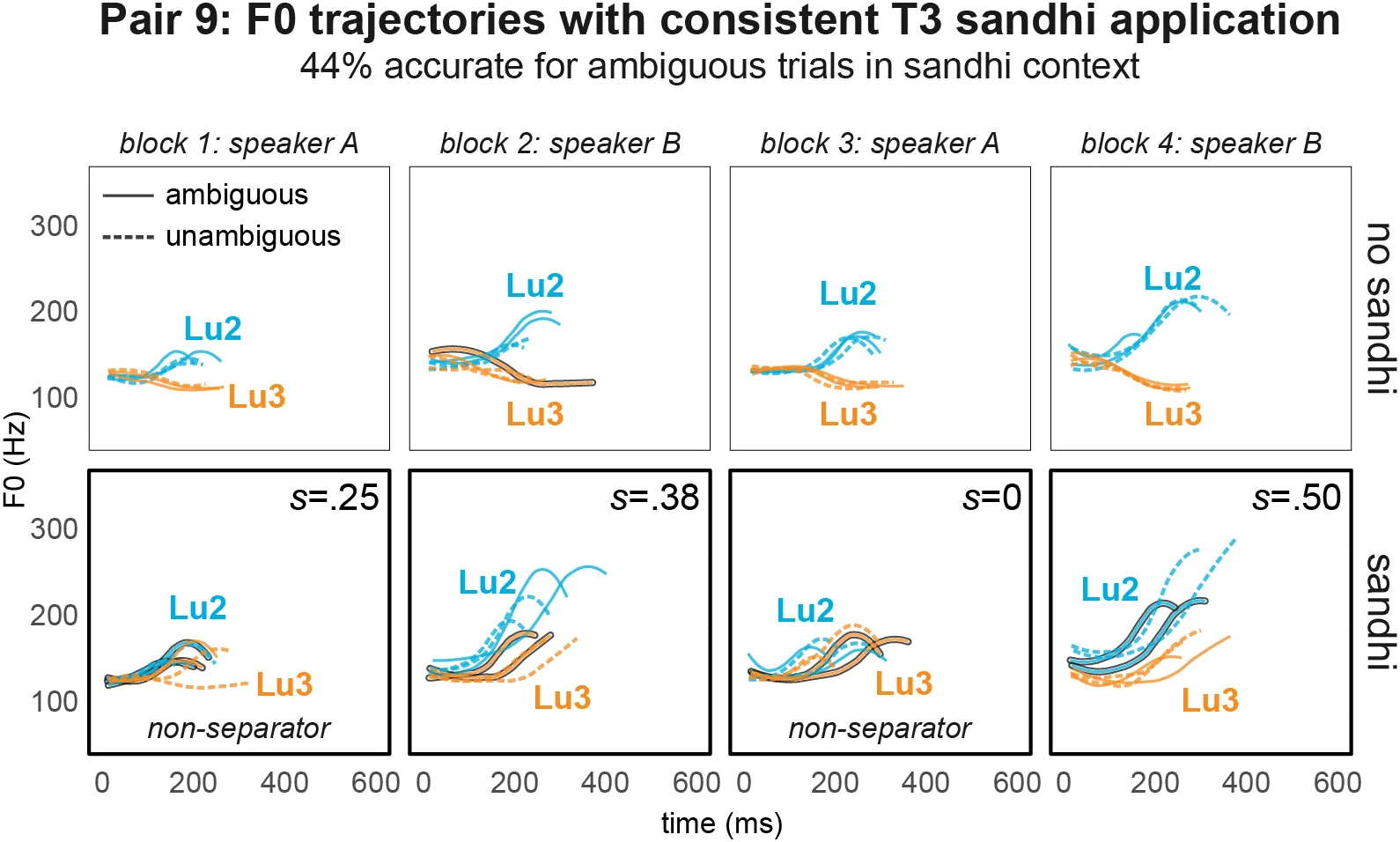
F0 trajectories for Lu2 and Lu3 in different sandhi contexts produced by Pair 9 across blocks of the Matching task. Misidentified tokens have a black outline.

Fig 6 illustrates the F0 trajectories for Lu2 and Lu3 produced by Pair 9. Visual inspection indicates that they consistently applied T3 sandhi, resulting in rising F0 trajectories (T3S) for 15 out of 16 Lu3 tokens in sandhi context. In the no sandhi context, there is clear separation between these surnames, with Lu3 having a consistent low trajectory without any rise (half T3) for all tokens. Visual inspection confirms that this half T3 trajectory was the norm for T3 in no sandhi context for nearly all speakers in all pairs. For Pair 9, this distinction between rising T2 and half T3 was almost completely lost in sandhi context, regardless of whether the competitor surname was ambiguous or not. Speaker A (a non-separator) did not appear to make any consistent distinction between Lu2 and Lu3. Not surprisingly, this pair had difficulty correctly identifying Lu2 (m = 50%) and Lu3 (m = 38%) targets in sandhi context when the competitor was potentially ambiguous. Another pair with similarly little Lu2/Lu3 differentiation was Pair 3 (overall accuracy for ambiguous trials 56%; Speaker A a consistent non-separator, Speaker B a non-separator in block 2).

**Fig 7:**
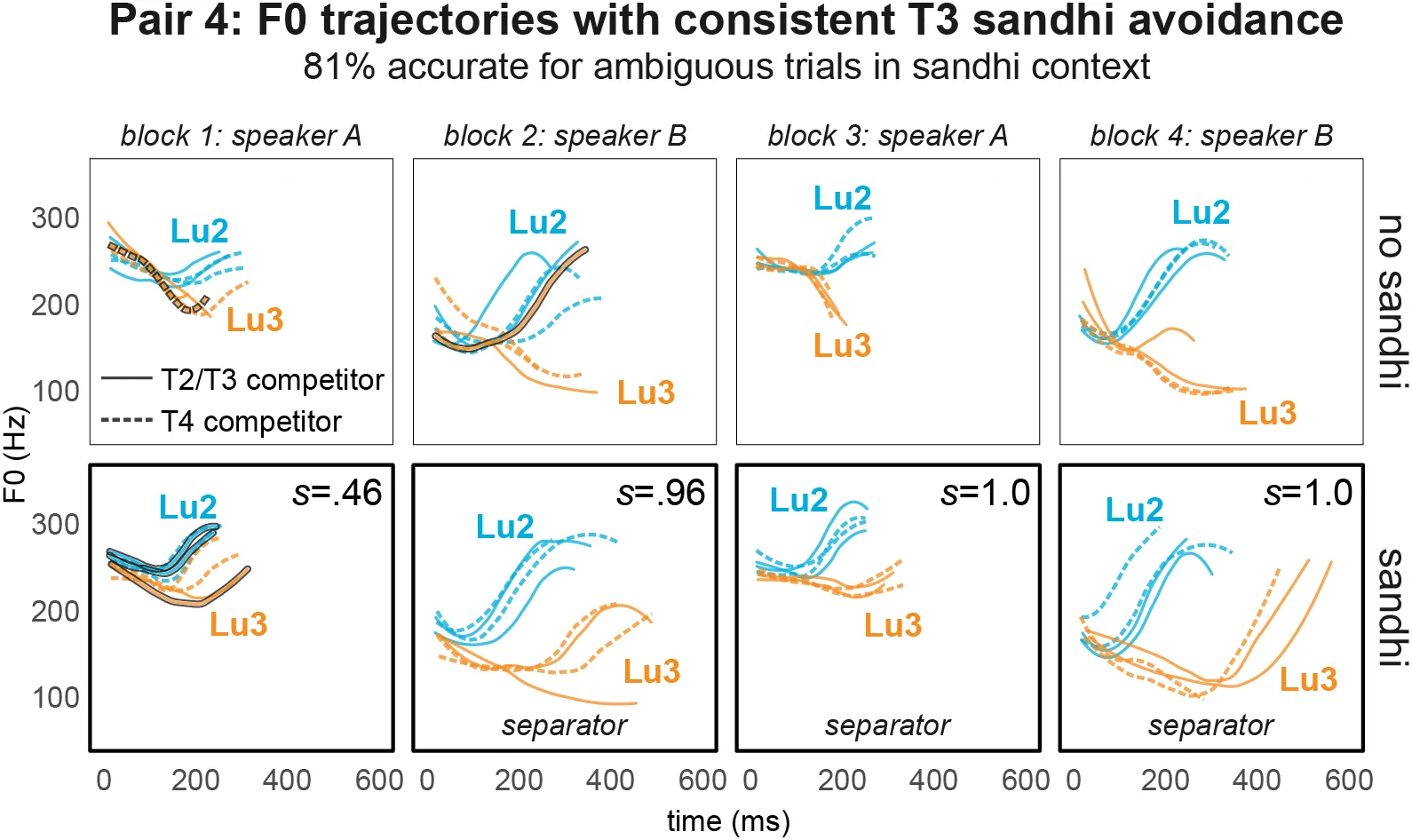
F0 trajectories for Lu2 and Lu3 in different sandhi contexts produced by Pair 4 across blocks of the Matching task. Misidentified tokens have a black outline.

In contrast to Pair 9, Pair 4 illustrates a more successful approach where Lu2 and Lu3 were consistently differentiated after the initial block. In Fig 7 we once again see clear differentiation of Lu2 and Lu3 in the no sandhi context for all blocks (with one stray Lu3 looking like T3S in Block 2). In sandhi context, however, we see consistently long and dipping full T3 trajectories that are typical pre-pausally, but not otherwise in connected speech. This might be interpreted as an example of sandhi avoidance. In Block 1, Speaker A applied T3 sandhi for Lu3 when the competitor was Lu4, but immediately began to produce low-dipping (non-sandhi) Lu3. In Block 2, Speaker B follows suit and for the rest of the sandhi context trials, T3 sandhi is never fully applied. This “sandhi avoidance”, whether accidental or intentional, can be observed in at least some trials from all pairs (even Pair 9). Pair 4 illustrates that this behavior is most often not simply a case of non-application of T3 sandhi—in that case, the F0 trajectory would be a half T3, just as in the no sandhi context. Instead, what we observe is that speakers produce dipping full T3 contours.

**Fig 8:**
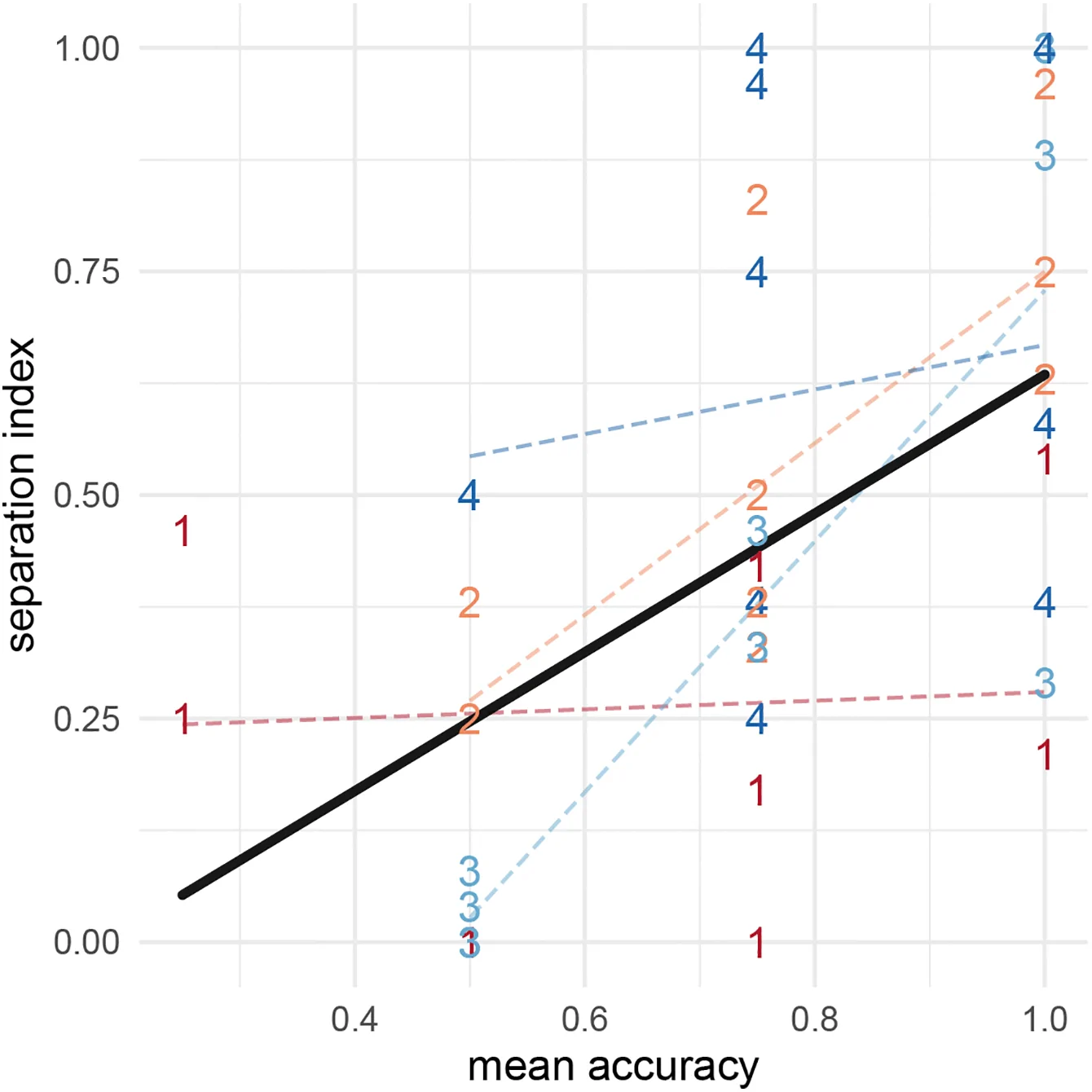
A scatterplot with regression lines depicting the possible relationship between listener’s identification accuracy for ambiguous trials, and speaker’s separation index. Colored numbers indicate each data point according the block (1, 2, 3, 4) of the experiment in which it occurred. The thick black line indicates the overall regression line for all data, colored dashed lines indicate the regression line for each block.

As the patterns we have identified with the separation index are post-hoc and exploratory, we did not attempt to incorporate them into our current quantitative models. However, we did explore the relationship between separation scores and response accuracy through correlational analysis and visualization (Fig 8). We conducted a Pearson’s product-moment correlation of the relationship between accuracy and the separation index. There was a moderate correlation, *r*(38) = .53, 95% CI [.26, .72], *p* < .001, indicating that increased separation was related positively to increased accuracy. We also examined the correlations by block; we note that there is very sparse data at this level of analysis. Correlations were strong in Block 2, *r*(8) = .73, 95% CI [.18, .93], *p* = .018, and Block 3, *r*(8) = .87, 95% CI [.53, .97], *p* = .001. In contrast, correlations in Block 1, *r*(8) =  .07, 95% CI [−.59, .67], *p* = .853, and Block 4, *r*(8) = .14, 95% CI [−.54, .71], *p* = .707, were weak. We tentatively interpret these correlational results as evidence of the meaningful relationship between a speaker’s separation of T2 and T3 and a listener’s ability to accurately identify those tones in sandhi context.

Though not observable in our figures, the production of a dipping T3 surname was sometimes accompanied by a pause before uttering the title. For example, Pair 2, speaker A, in Block 3 produced noticeable pauses in multiple utterances. In the post experiment survey, this participant explicitly noted inserting a pause as a strategy to avoid ambiguity (e.g., “[I] read the two consecutive Tone 3 words separately” “分开读这两个连在一起的第三声的词”). A second participant (from Pair 10) mentioned what might be a pausing strategy (“Separating the words [you] want to say will reduce ambiguity” “将要说的词分割开来说会减小歧义”), however, pauses are not evident in the recordings from Pair 10, nor are low-dipping T3 trajectories (see S7 Fig in the Supporting Information).

One question that cannot be answered from any of the acoustic or auditory data is the extent to which speaker’s T3 or T2 adjustments were intentional. The post-experiment survey provides evidence that at least some participants were consciously aware of the ambiguity affecting Lu2 and Lu3. In response to the question: “Just now when you were completing tasks as a pair, did you notice anything?” Half (10/20) of the participants indicated difficulty related to tones, surnames, or homophones, with several noting Lu2 and Lu3 specifically as the issue. Further interrogation along these lines suggests that many of those who noticed the issue also tried to solve it, though the specificity of their explanation varied. In response to the question of how they tried to resolve the problem, one person (from Pair 4) wrote that they solved the problem by “using more force for Tone 3”, another (from Pair 9) “by reading the tones more accurately,” and yet another (from Pair 5) simply “by using tones.” When asked to provide examples of tone changes that occur in Mandarin, 9 of 20 participants provided examples of T3 and T2 surnames. When asked how they knew, many indicated it was something they learned in school, some that it was “native speaker knowledge”, and a couple that it was something they learned from doing the experiment.

## Discussion

Given the preponderance of evidence that T3 sandhi leads to tonal ambiguities, we investigated how Mandarin speakers adjust their speech production to overcome potential ambiguities induced by T3 sandhi during a structured interactive task. Results indicated that sandhi context induced some perceptual ambiguity for surnames with T3, leading to confusion between a surname with T2 (Lu2) and one with underlying T3 (Lu3). However, most pairs of participants were able to navigate these difficulties by making adjustments to their production of T3 and T2 in sandhi context. These adjustments would not be observable in typical solo context tasks, and provide evidence of the potential utility of interactive tasks such as we used in this study. Below, we elaborate on some of the insights gained from the present study.

Already in the 1960s, Wang and colleagues [4] and Wang and Li [5] provided evidence that T3 sandhi could induce native Mandarin speakers to experience perceptual ambiguities. They found listeners performing at chance when identifying recordings of two-syllable phrases contrasting T2-T3 vs T3-T3 sequences. The present study provides further evidence of perceptual ambiguity caused by T3 sandhi, showing that it can happen in actual interactions, and that interlocutors might not be able to fully avoid it even when they are trying.

At the same time, our data show that people can and do adjust their T3 production in ways that might reduce ambiguity. Through exploration of individual speaker F0 trajectories and auditory files, we tentatively identified two adjustments: producing lowering T3 F0 towards the end of the trajectory (sandhi avoidance) and adding pauses. Elicitation of these adjustments was only possible through the use of a task that provided for both communicative interaction and allowed only acoustic modifications. Without communication, speakers might not notice the ambiguities. Without control over their choices in how to navigate the task, they might have chosen alternative strategies such as describing the written Chinese form of the intended surname.

Overall GAMM results indicated that speakers may have produced T3 in sandhi context with a lower F0 rise compared to how they produced T2. This would be consistent with findings from previous research [2,10–13]. However, both our second GAMM (for ambiguous trials only) and qualitative examination of individual T3 productions caution against taking this difference at face value. At least for the current data, it seems that the average of T3 in sandhi context in the overall GAMM analysis reflects an artefactual pattern where the average height of T3S has been pulled down by productions that avoided application of T3 sandhi altogether. We argue that what that model presents is not T3S, but an average of qualitatively different T3 allotones that includes T3S, but also full T3, and half T3. This makes it impossible to determine whether T3S productions are in fact lower than T2, or if the apparently lower F0 trajectory is simply an artefact of averaging. At the same time, our second GAMM analysis targeting only ambiguous trials did provide some support for a lower T3S trajectory (with incorrectly identified T3 surnames) compared to T2. In sum, we suggest these findings should temper interpretations of previous results that take at face value the acoustic differences between T2 and T3S, as the variant forms of T3 may not be accurately captured by processes that rely only on averaging.

Our second GAMM analysis indicated that, for trials where Lu2 and Lu3 were likely to become ambiguous if T3 was applied, correctly identified T3 tokens tended to have a lower F0 in the latter portion of the trajectory. Based on qualitative exploration, we have suggested this lower F0 reflects sandhi avoidance, with T3 realized as either a half-T3 or full-T3, rather than as T3S. Future research will be needed to directly test this hypothesis. What the present data show clearly is that when T3 had a higher F0 trajectory (more similar to canonical T2), misidentification of T3 as T2 increased, despite the average late F0 trajectory of these T3s still being significantly lower than T2 (Fig 4, Panel C). This pattern provides some further context for considering the observation made by Yuan and Chen [13] that, because of substantial overlap among F0 trajectories for T3S and T2, even if there are differences in their F0 rise, this difference alone will not be sufficient to help listeners in most cases.

A novel and unexpected finding was that our overall GAMM indicated that some speakers adjusted to sandhi context by raising the F0 offset height of T2. One reviewer noted that this pattern could be due (at least in part) to anticipatory coarticulation related to the different titles used in the two contexts. This is a reasonable explanation for this effect in the overall GAMM. Furthermore, our second GAMM examining only the trials in ambiguous sandhi contexts did not show evidence of T2 differences. We therefore believe the reviewer’s explanation is likely correct, and have not pursued a strong interpretation of the possible differences between T2 productions in the present study.

### Limitations and future directions

Although this study identified a realistic context where ambiguity could arise, one obvious limitation for how results generalize is the strongly controlled nature of the communicative task. Outside of this task, speakers would have additional freedom to choose non-acoustic strategies to avoid ambiguity. Future work could deploy additional tasks that allow for such freedom, as well as controlled tasks that do not include a communicative goal, in order to allow comparisons of how the same set of speakers produce tones under different conditions.

We also note that our dataset is relatively small, with only 20 speakers (10 interlocutor pairs). Future work might aim to collect data from more speakers, more tokens from each speaker, and a greater variety of surnames (or other targets for T3 sandhi).

We tentatively identified two articulatory strategies that participants seem to have utilized during the matching task (lowering T3 F0, pausing). We have tried not to speculate about how intentional such strategies were, except where warranted by participant comments. Future work could investigate these strategies in a more systematic way, and determine more clearly if they are intentional or not, and used consistently, or not.

One aspect of the current data that we did not explore is creakiness, which is typically associated with T3 (or any low F0 trajectory, cf. [11]). Our present analysis did not account for the possible role creakiness might play in disambiguating T2 and T3 in sandhi contexts. Future research might address this issue directly.

We finish by noting that the approach we use here has potential practical applications. As a result of our controlled task, we were able to observe that some acoustic adjustments (lowering T3 F0) are more effective for resolving ambiguity. With further refinements to the study design, such insights could give us confidence to provide guidance for people (or machines) on the best strategies for avoiding or resolving ambiguities.

## Supporting information

S1 Fig F0 trajectories for Lu2 in no sandhi and sandhi contexts produced by Pair 2 across blocks of the Matching task. (TIF)

S2 Fig F0 trajectories for Lu2 in no sandhi and sandhi contexts produced by Pair 3 across blocks of the Matching task. (TIF)

S3 Fig F0 trajectories for Lu2 in no sandhi and sandhi contexts produced by Pair 5 across blocks of the Matching task. (TIF)

S4 Fig F0 trajectories for Lu2 in no sandhi and sandhi contexts produced by Pair 6 across blocks of the Matching task. (TIF)

S5 Fig F0 trajectories for Lu2 in no sandhi and sandhi contexts produced by Pair 7 across blocks of the Matching task. (TIF)

S6 Fig F0 trajectories for Lu2 in no sandhi and sandhi contexts produced by Pair 8 across blocks of the Matching task. (TIF)

S7 Fig F0 trajectories for Lu2 in no sandhi and sandhi contexts produced by Pair 10 across blocks of the Matching task. (TIF)
